# Microbial Sources of Exocellular DNA in the Ocean

**DOI:** 10.1128/aem.02093-21

**Published:** 2022-03-21

**Authors:** Morgan D. Linney, John M. Eppley, Anna E. Romano, Elaine Luo, Edward F. DeLong, David M. Karl

**Affiliations:** a Department of Oceanography, Daniel K. Inouye Center for Microbial Oceanography: Research and Education (C-MORE), University of Hawai‘i at Mānoa, Honolulu, Hawai‘i, USA; Norwegian University of Life Sciences

**Keywords:** bacteriophage, bacterioplankton, eDNA, exocellular DNA, free DNA, metagenomics, microbial ecology, microbial oceanography, open ocean, vesicle

## Abstract

Exocellular DNA is operationally defined as the fraction of the total DNA pool that passes through a membrane filter (0.1 μm). It is composed of DNA-containing vesicles, viruses, and free DNA and is ubiquitous in all aquatic systems, although the sources, sinks, and ecological consequences are largely unknown. Using a method that provides separation of these three fractions, we compared open ocean depth profiles of DNA associated with each fraction. *Pelagibacter*-like DNA dominated the vesicle fractions for all samples examined over a depth range of 75 to 500 m. Viral DNA consisted predominantly of myovirus-like and podovirus-like DNA and contained the highest proportion of unannotated sequences. Euphotic zone free DNA (75 to 125 m) contained primarily bacterial and viral sequences, with bacteria dominating samples from the mesopelagic zone (500 to 1,000 m). A high proportion of mesopelagic zone free DNA sequences appeared to originate from surface waters, including a large amount of DNA contributed by high-light *Prochlorococcus* ecotypes. Throughout the water column, but especially in the mesopelagic zone, the composition of free DNA sequences was not always reflective of cooccurring microbial communities that inhabit the same sampling depth. These results reveal the composition of free DNA in different regions of the water column (euphotic and mesopelagic zones), with implications for dissolved organic matter cycling and export (by way of sinking particles and/or migratory zooplankton) as a delivery mechanism.

**IMPORTANCE** With advances in metagenomic sequencing, the microbial composition of diverse environmental systems has been investigated, providing new perspectives on potential ecological dynamics and dimensions for experimental investigations. Here, we characterized exocellular free DNA via metagenomics, using a newly developed method that separates free DNA from cells, viruses, and vesicles, and facilitated the independent characterization of each fraction. The fate of this free DNA has both ecological consequences as a nutrient (N and P) source and potential evolutionary consequences as a source of genetic transformation. Here, we document different microbial sources of free DNA at the surface (0 to 200 m) versus depths of 250 to 1,000 m, suggesting that distinct free DNA production mechanisms may be present throughout the oligotrophic water column. Examining microbial processes through the lens of exocellular DNA provides insights into the production of labile dissolved organic matter (i.e., free DNA) at the surface (likely by viral lysis) and processes that influence the fate of sinking, surface-derived organic matter.

## INTRODUCTION

Over half of the DNA in the open ocean is present outside living organisms (1–4). The DNA that passes through a membrane filter (0.1 or 0.2 μm) is exocellular (also termed “dissolved” DNA [5]) and is comprised of three known pools: protein-encapsidated viruses, exocellular vesicles, and free DNA. The cycling of labile exocellular DNA in the ocean has consequences for global nutrient cycling, export, and potential for genetic exchange. DNA in the ocean has been studied from multiple oceanographic perspectives (e.g., studying environmental DNA [also termed “eDNA”] for fishery management, sequencing microbial cellular and free DNA to identify taxonomic relationships and quantify population diversity, and quantifying viral diversity and dynamics), making the overlapping terminology challenging to disentangle. While eDNA research may have similar objectives of holistically studying an ecosystem, by collecting the remnants of large macroorganisms (e.g., scales, mucus, and reproductive cells) to uncover which organisms are present in a given study site (6), it employs the same collection method as that used for cellular analyses (7). DNA such as that which is captured on a filter is equivalent to “particulate” DNA (2), distinguishing it from DNA that passes a filter (exocellular DNA).

Subtropical gyres comprise 40% of the Earth’s surface, yet surface waters are depleted of essential elements like nitrogen and phosphorus (8), highlighting exocellular DNA (with a C-to-N-to-P molar ratio of 10:4:1) as a nutrient-rich molecule in this system. It can be taken up as polydeoxyribonucleotides (>10 kb) (9–11) as well as hydrolyzed and broken down to nucleotides and nucleic acid bases (12–14). Alternatively, the genomic information encoded by exocellular DNA can be integrated by natural transformation (15).

One of the largest ecosystems on Earth, the North Pacific Subtropical Gyre (15°N to 35°N latitude and 135°E to 135°W longitude) is also one of the best-studied water columns (8). Thirty years of biogeochemical research provide a wealth of baseline data sets: hydrographic observations (and their implications for thermohaline circulation), metabolic balances, nutrient cycling, cultured microbial isolates, complex microbial interactions, and export dynamics. More recently, gene catalogs of cellular and viral communities throughout the water column at Station ALOHA have broadened our understanding of the distributional patterns of diverse microbial and viral communities in the open ocean (7).

Recently, exocellular vesicles from abundant ocean microorganisms such as *Prochlorococcus* (16), *Synechococcus*, and Pelagibacter ubique (17) have also been documented as components of exocellular DNA in the open ocean. These lipid-enclosed structures can contain a variety of proteins and nucleic acids (DNA and RNA). However, the function and dynamics of these structures in the open ocean are still unclear, but they are hypothesized to be decoys for viral lysis (16) and possibly a mode of mobile gene transfer (18).

While vesicles and viruses, two of the three exocellular DNA constituents, have been examined previously in open ocean systems, the biological composition and depth origins of free DNA are relatively uncharacterized. This limitation is primarily due to challenges presented in separating exocellular DNA fractions and isolating free DNA. For instance, exocellular DNA (described previously as dissolved DNA) isolation methods utilized low-volume filtration (19) or chemical precipitation (5, 20) to isolate bulk exocellular DNA. These previous methods pose challenges in acquiring enough free DNA (>0.1 μg) to identify the microbial sources by metagenomic sequencing. Together, exocellular DNA comprises nearly half of the DNA inventory in the open ocean, with the particulate (detrital and cellular) DNA comprising the other half (2, 21, 22). Compositionally, the average proportions of exocellular DNA fractions (free DNA, viruses, and vesicles) are 45, 35, and 20%, respectively (22); this leaves at least 25% of the open ocean DNA inventory uncharacterized (21, 22).

To investigate all three exocellular DNA pools and their microbial sources, we employed a method (22) that separates the three pools by density. DNAs from vesicle, virus, and free DNA fractions were sequenced and compared. This study provides the first genetic characterization of the free DNA pool, alongside the other exocellular DNA pools, and reveals viral lysis as a possible source of free DNA and export as a mechanism of delivery of DNA to the mesopelagic zone (250 to 1,000 m).

## RESULTS

### Habitat structure.

Exocellular DNA samples were collected on three cruises in the North Pacific Subtropical Gyre to Station ALOHA (22°45′N, 158°W): (i) November 2017, HOT-297 (see Table S1 and Fig. S1 in the supplemental material); (ii) April 2018, FK180310; and (iii) May 2018, HOT-302. For these cruises, inorganic nutrients (nitrate plus nitrite [N+N] and phosphate) were typical of conditions at Station ALOHA in the euphotic zone (0 to 200 m), ranging from <0.1 to 3.3 and <0.1 to 0.3 μM (N+N and phosphate, respectively). In the lower mesopelagic zone (500 to 1,000 m) values ranged from 32 to 42 and 2.4 to 3.0 μM (N+N and phosphate, respectively). Fluorometric chlorophyll *a* ranged from <0.1 to 0.3 μg L^−1^ in the euphotic zone, consistent with historical averages. In the upper euphotic zone (5 to 100 m), *Prochlorococcus* cell abundances were consistent with previous Station ALOHA measurements (1.1 × 10^5^ to 1.8 × 10^5^ cells mL^−1^) and declined sharply (<0.1 × 10^5^ to 0.3 × 10^5^ cells mL^−1^) in the lower euphotic zone (125 to 175 m). Cell abundances of heterotrophic bacteria were consistent with historical euphotic ranges (1.8 × 10^5^ to 5.0 × 10^5^ cells mL^−1^).

### Apparent microbial sources of free DNA and other exocellular DNA pools. (i) Sources of free DNA.

**FIG 1:**
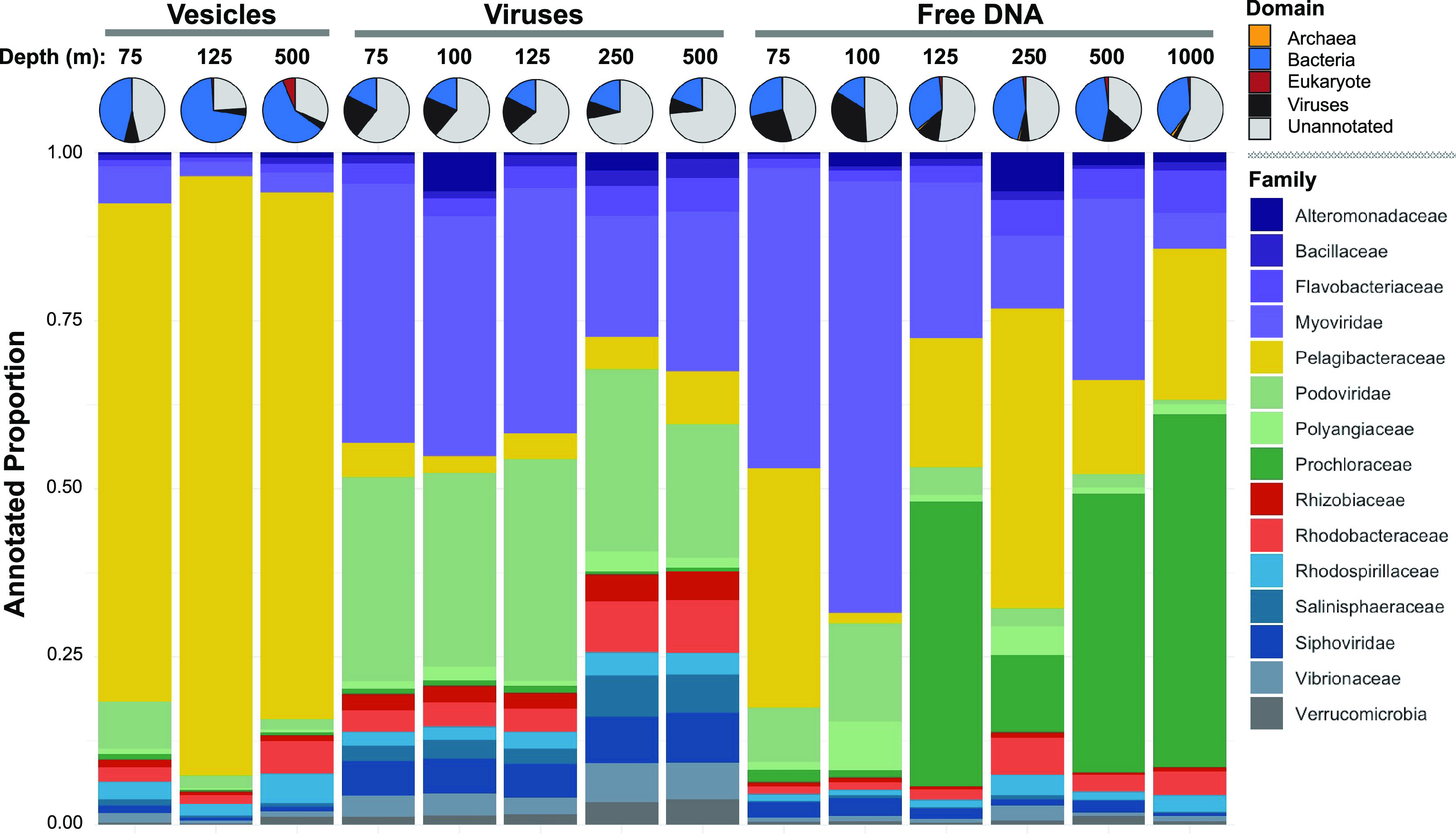
Taxonomic annotation of metagenomic sequences from the three exocellular DNA constituents collected from the North Pacific Subtropical Gyre. Pie charts along the top display the domain-level taxonomic composition and proportion of unannotated sequences for each sample. Stacked bar charts represent the family-level taxonomic compositions of the three isolated exocellular DNA constituents (vesicles, viruses, and free DNA). The vesicle constituents were collected from three euphotic and mesopelagic zone depths (75, 125, and 500 m) in the North Pacific Subtropical Gyre. Viral fractions were collected from five depths (75, 100, 125, 250, and 500 m). Free DNA samples were collected from six depths (75, 100, 125, 250, 500, and 1,000 m).

Summed across six free DNA samples collected in the North Pacific Subtropical Gyre (DNA concentrations ranging from 0.1 to 0.15 μg L^−1^) (Fig. S2), the majority of annotated genes in the DNA-based metagenomic libraries were derived from bacteria and viruses (Fig. 1), with lesser contributions from eukaryotes and archaea (<1.0 and 1.1% of all annotated sequences, respectively). The proportion of viral free DNA sequences in surface waters ranged from 10 to 35% of annotated sequences above or at the deep chlorophyll maximum (DCM) (75 to 125 m), about the same as the proportion of bacteria (16 to 34%) over the same depth range. However, in the mesopelagic zone (250 to 1,000 m), the proportion of annotated free DNA sequences from viruses was lower (2 to 16%), whereas the proportion of bacterium-derived sequences was higher (38 to 45%). Across all free DNA metagenomic libraries, the biological composition was dominated (69 to 85% of family-level-annotated sequences) by three main taxonomic groups: *Pelagibacteraceae*, *Myoviridae*, and *Prochlorococcus* (Fig. 1), all of which have been documented as abundant sources of DNA in the North Pacific Subtropical Gyre (23–26). Ubiquitous heterotrophs of the family *Pelagibacteraceae* contributed an average of 23% of annotated free DNA sequences over all depths sampled, with a range of 2 to 46%. *Prochlorococcus* represented 43, 42, and 53% of free DNA sequences collected from 125, 500, and 1,000 m, respectively, and represented an average of 23% of family-level-annotated sequences. Of these *Prochlorococcus* sequences (125, 500, and 1,000 m), more than 85% were from the high-light (HL) ecotypes (Table S3). The highest proportions of *Myoviridae*-annotated free DNA sequences were observed in the upper euphotic zone, 45% (75 m) and 69% (100 m). Of the virus-derived sequences constituting the free DNA samples, the majority (>90% of annotated viral sequences with known hosts) were dominated by viruses known to infect *Pelagibacter* (16% ± 7.2%), *Synechococcus* (38% ± 4.6%), *Prochlorococcus* (31% ± 4.8%), and other cyanobacteria (7.9% ± 1.3%) (Fig. S3). *Synechococcus* viruses peaked at the DCM on two occasions (43%). Other viruses known to infect SAR116 (5.4% ± 2.2%) and *Vibrio* (1.1% ± 0.6%) were detected at much lower proportions in the free DNA. The proportions of free DNA-derived *Vibrio* sequences were highest in the mesopelagic zone (250 m, 2.3%; 500 m, 1.0%; 1,000 m, 1.4%) but were only <1% in the euphotic zone samples.

Among other taxonomic groups contributing to metagenomic free DNA libraries, the archaeal contribution was the smallest (ranging from 0.2 to 2.0% of annotated sequences for all samples). Of these, they were most similar to ammonia-oxidizing *Nitrosopumilaceae* (<0.1 to 1.6%), which were most prevalent at depths >100 m. Of the three domains contributing to free DNA metagenomes, eukaryotes comprised the lowest proportion (0.3 to 1.5% of annotated free DNA). The taxonomic families that were the most abundant included heterokont Pelagomonadaceae, coccolithophorid Noelaerhabdaceae, and Bathycoccaceae.

### (ii) Sources of vesicle DNA.

Exocellular vesicle DNAs (DNA concentrations ranging from 0.03 to 0.10 μg L^−1^) (Fig. S2) collected throughout the euphotic and mesopelagic zones (75 to 500 m) (Fig. 1) were also sequenced, compared, and microscopically assessed by transmission electron microscopy (Fig. S2). Summed across all samples, bacteria contributed overwhelmingly to these metagenomic libraries (77% ± 22% of all annotated sequences). Viruses, archaea, and eukaryotes contributed averages of 18%, 3.7%, and 0.6%, respectively, to all annotated sequences (Table S2). The presence of viral, archaeal, and eukaryotic DNA in the vesicle fraction in addition to bacterial sequences is consistent with previous open ocean investigations of vesicle DNA (16). At the family level, bacterial sequences were dominated by *Pelagibacteraceae* (81% ± 8% of family-level-annotated sequences), with only 34% ± 12% of domain-level sequences being left unannotated, the lowest of all three exocellular DNA fractions. Other bacterial sequences that contributed to the annotated vesicle samples include those from *Rhodospirillaceae* (2.9%), *Rhodobacteraceae* (2.7%), *Flavobacteriaceae* (1.0%), and *Prochlorococcus* (0.5%). Viral sequences in the vesicle metagenomic DNA were minimal but, when present, were comprised of podoviruses (7.0%) and myoviruses (6.0%).

### (iii) Sources and putative hosts of the viral exocellular DNA fraction.

Averaged across five virus fraction metagenomic libraries (DNA concentrations ranging from 0.05 to 0.14 μg L^−1^) (Fig. S2), the exocellular DNA samples were dominated by annotated sequences derived from viruses (Fig. 1), consistent with previous reports of this exocellular DNA fraction that utilized transmission electron and epifluorescence microscopic analyses (22). The viral metagenomic libraries had the lowest number of recovered sequences, many of which were novel and unannotated (61 to 73% unannotated across all samples). For all samples, viral family-level-annotated sequences were nearly split between myoviruses (23 to 38%) and podoviruses (19 to 32%), with minimal contributions from siphoviruses (4 to 7%). Of the metagenomic libraries contributing to the viral libraries, *Prochlorococcus* phages, other cyanophages, and *Pelagibacter* phages were the most abundant (Fig. S3). *Synechococcus* and *Prochlorococcus* phages dominated the euphotic samples (75 to 125 m). At the DCM, the relative read abundances of *Synechococcus* phage and cyanophage sequences in the virus exocellular DNA fraction peaked, whereas in mesopelagic zone samples, *Pelagibacter* phages and *Vibrio* phages increased in proportion. These depths are consistent with both cellular host (0.2 μm filtered) as well as free virus (0.02 μm filtered) abundances previously reported at Station ALOHA (23, 25–27).

Overall, the metagenomic libraries developed from the three exocellular DNA pools were distinct with respect to their microbial DNA compositions. The vesicle fraction was primarily dominated by a single taxonomic family (*Pelagibacteraceae*) across all depths, the viral fraction was dominated by bacteriophages, and, finally, the exocellular free DNA pool had both bacterium- and virus-derived DNA. While DNAs from the former two pools have been previously described by metagenomic analyses, the composition of the free DNA fraction has not been previously reported.

### Estimating the depth of origin for exocellular DNA throughout the water column.

**FIG 2:**
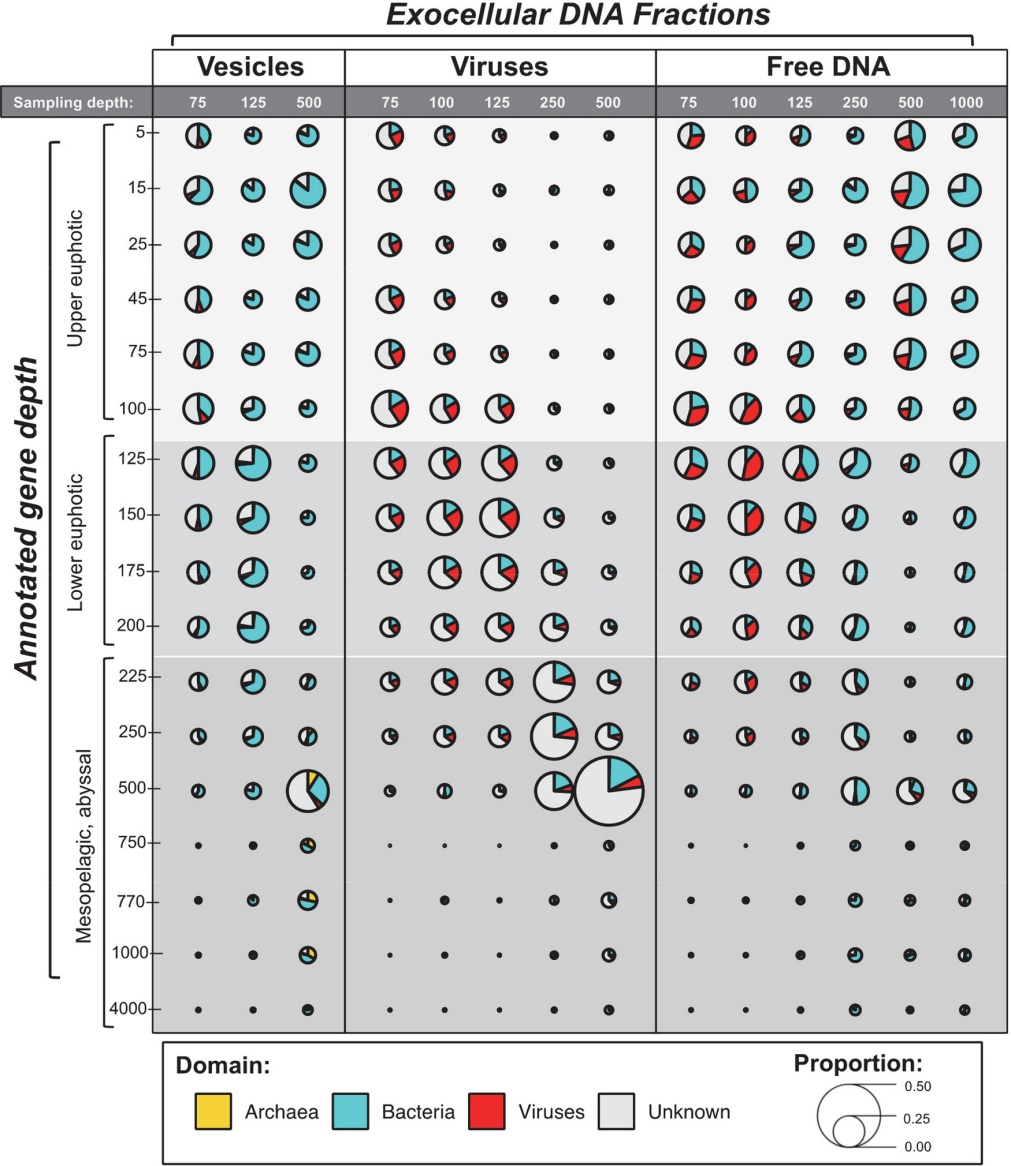
Annotation of exocellular DNA metagenomic sequences suggests their probable depths of origin. The best sequence matches to the ALOHA 2.0 gene catalog (and their corresponding sampling depths) were used to assign the probable depth of origin to individual exocellular DNA metagenomic sequence reads. The size of the pie chart is proportional to the total number of exocellular DNA metagenomic reads, whose best match is to a Station ALOHA gene originating from a given corresponding water depth. Eukaryotes are excluded due to a lack of visibility, and all pie chart proportions are <0.004 for all samples and depths.

To infer the depths of origin of different exocellular DNA fractions, we mapped the DNA sequences against a depth-resolved microbial gene catalog (ALOHA 2.0) isolated from Station ALOHA (Fig. 2) (7, 26) that includes both cellular and viral sources. The objective was to determine whether genes from the exocellular DNA fractions matched ALOHA 2.0 genes recovered from the same sampling depths as those for the exocellular DNA or whether the exocellular DNA was potentially transported from other depths or regions.

The viral exocellular DNA samples isolated in this study were most similar in identity and depth resolution (i.e., the depth from which the viral exocellular DNA sample was collected in this study was consistent with the collection depth of the mapped ALOHA 2.0 catalog genes). This was particularly evident in the mesopelagic zone samples collected from 250 to 500 m (Fig. 2). In these samples, 25% of the viral exocellular DNA genes were derived from their respective collection depths, with only 13% being derived from the euphotic zone (5 to 200 m). Viral samples collected from the DCM had high contributions from typical DCM depths (100 to 175 m) (29 to 36%) as well as neighboring upper euphotic (5 to 75 m) (15%) and upper mesopelagic (200 to 250 m) (13%) zones, with only 1% being from the lower mesopelagic zone (500 to 1,000 m). Of the three exocellular DNA fractions, the viral samples had the highest average percentage of genes originating from unassigned depths (43% ± 4.5%).

In contrast to the viral samples, the vesicle and free DNA samples appeared to contain both autochthonous and allochthonous DNA (Fig. 2). In the euphotic zone samples (75 to 125 m), as expected, sequences were dominated (>50%) by surface-derived DNA (5 to 200 m), with minimal mesopelagic zone contributions (<10%). However, in mesopelagic zone vesicle and free DNA samples (250 to 1,000 m), genes originated primarily from the upper euphotic zone (5 to 75 m) (>30%) and, to a lesser extent, the depth from which they were collected (<20%). Of these mesopelagic zone samples, the shallowest free DNA sample (250 m) had the most genes that apparently originated from multiple depths throughout the euphotic and mesopelagic zones (5 to 500 m).

### Size distributions of environmental free DNA through the water column.

**FIG 3:**
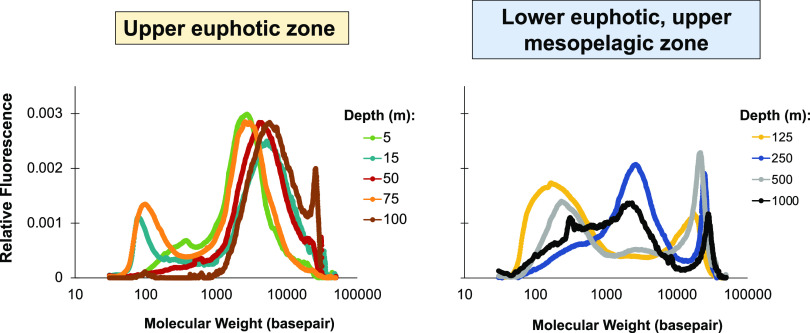
Fragment analysis of free DNA samples from the North Pacific Subtropical Gyre. The relative fluorescence units of each sample were used to normalize and calculate proportions. Molecular weight values are shown from 75 to 50,000 bp according to the Agilent DNF-488-0500 protocol sizing range. Samples collected above the deep chlorophyll maximum (125 m) had more distinct peaks and less DNA outside the peaks, while samples below had a broader distribution of sizes, indicative of degradation.

The size spectra of recovered free DNA were measured by capillary electrophoresis (CE), following density gradient separation and buffer exchange. Seven free DNA samples collected throughout the euphotic and mesopelagic zones (5 to 1,000 m) were measured to assess nucleic acid quality, size ranges, and potential degradation artifacts, prior to sequencing (Fig. 3). Previous work (22) evaluated the effects of the method on different lengths (75 to 20,000 bp DNA ladder) of microbial DNA. This work found that there was no discernible shearing of all DNA sizes and concentrations (0.2 to 1 μg L^−1^) tested, with 65 to 80% of the DNA being recovered.

In this study, samples collected in the upper euphotic zone (5 to 100 m) had a distinct peak (<5,000 bp peak width) of longer, high-molecular-weight (HMW) free DNA (referred to here as >1,000 bp) and a lower proportion of shorter, low-molecular-weight (LMW) (<1,000 bp) free DNA, ranging between 24 and 38%, compared to mesopelagic zone (250 to 1,000 m) samples, which ranged between 33 and 65%. In the upper euphotic zone free DNA samples, there was a high proportion of 1,000 to 40,000 bp DNA (62 to 73%), whereas in mesopelagic zone samples, the proportion of this HMW free DNA tended to be lower (48% on average [range, 35 to 66%]). Lower euphotic zone (125 m) and mesopelagic zone (250 to 1,000 m) samples tended to have a broader range of free DNA sizes (base pairs), suggesting that this DNA may have been more degraded. In mesopelagic zone samples, the HMW free DNA decreased, the maximum peaks of HMW DNA in samples from 250 to 1,000 m were less distinct (>10,000 bp peak width), and there was more free DNA between peaks. In samples from 5 to 100 m, <25% of the DNA was <350 bp. At 1,000 m, the peaks were unpronounced, suggesting a notable level of degradation in this deep sample.

### Comparison of vesicle, viral, and free DNA fractions by nonmetric multidimensional scaling.

To compare all exocellular DNA fractions (vesicles, viruses, and free DNA) with each other and previously reported Station ALOHA viral and cellular metagenomic sequences, two-dimensional ordination methods were employed. Bray-Curtis dissimilarity-based nonmetric multidimensional scaling (NMDS) data for the family-level-annotated metagenomes of all exocellular DNA fractions were compared with those of both the viral DNA (0.02 μm filtered) (Fig. S4A) and particulate DNA (0.2 μm filtered) (Fig. S4B) samples from the Station ALOHA gene catalog. All pairwise comparisons were analyzed by permutational multivariate analysis of variance (PERMANOVA) (ADONIS) using Benjamini-Hochberg correction for multiple comparisons. These analyses confirmed that the viral samples (viral exocellular DNA) collected in this study were similar in composition to previously characterized Station ALOHA virioplankton communities recovered from the same respective depths (Fig. S4A) (stress = 0.11), with no significant differences being identified (*P* value of >0.1 by PERMANOVA) for all depths (75 to 1,000 m). As for free DNA sequences, upper euphotic zone free DNA samples (75 to 100 m) clustered with catalog viral samples collected from the same depths, with no significant differences being identified (*P* value of >0.1 by PERMANOVA). However, free DNA samples collected at the deep chlorophyll maximum and below (125 to 1,000 m) clustered together and were significantly different from catalog viral samples from the same depths (*P* value of <0.01 by PERMANOVA). Similarly, vesicle DNAs did not cluster with any Station ALOHA viral samples and were significantly different from all catalog viral samples (*P* value of <0.01 by PERMANOVA).

The same exocellular DNA sequences were compared to the cellular microbial community genes by utilizing NMDS (stress = 0.07) (Fig. S4B). From this analysis, two distinct cellular communities emerged (*P* value of <0.01 by PERMANOVA): euphotic zone (75 to 125 m) and mesopelagic zone (250 to 1,000 m) Station ALOHA samples clustered together, and were consistent with previous reports (23, 25). Viral exocellular DNA did not cluster with any Station ALOHA cellular communities and were significantly distinct (*P* value of <0.01 by PERMANOVA). Similarly, free DNA fractions did not cluster with their respective Station ALOHA depths (*P* value of <0.01 by PERMANOVA), and deep free DNA samples (500 and 1,000 m) clustered with cellular communities filtered from 75 m, with no significant differences being identified (*P* value of >0.1 by PERMANOVA), revealing a potential origin of this free DNA. Upper mesopelagic zone (250 m) free DNA clustered nearest cellular metagenomes collected from 125 m and was not significantly different (*P* value of >0.1 by PERMANOVA). Surface (75 m) vesicle DNA clustered with euphotic zone cellular communities, whereas lower euphotic (125 m) and mesopelagic (500 m) zone vesicle samples clustered with mesopelagic zone (250 to 1,000 m) Station ALOHA cellular samples and were not significantly different (*P* value of >0.1 by PERMANOVA). Exocellular DNA samples that had high proportions of viral sequences (Fig. 1) clustered together (Fig. S4B) (viral exocellular DNA, 100-m free DNA, and unseparated exocellular DNA from 100 and 250 m).

## DISCUSSION

The identification of the sources and sinks of exocellular DNA in the ocean is important in order to better understand the diversity of marine life and microbial dynamics. Here, we show that exocellular DNA is comprised of three known pools: vesicles, viruses, and free DNA. Recent work characterized viral communities found throughout the water column at Station ALOHA (26–28) as well as vesicles as a potential mode of mobile gene element transfer (18). The vesicle fraction was anticipated to be well represented by DNA from the cyanobacterium *Prochlorococcus* (16, 29), yet *Prochlorococcus* appeared to contribute only 0.5 to 1% of the annotated sequences. Instead, the vesicle-derived DNA sequences were dominated by DNA derived from the ubiquitous heterotrophic bacterial family *Pelagibacteraceae*. The predominance of *Pelagibacter* in the vesicle pool may have several explanations. This DNA fraction could be derived, in part, from intact ultrasmall *Pelagibacter* cells (<0.1 μm) or from *Pelagibacter* vesicles. It was recently documented that structures resembling vesicles were produced by *Pelagibacter* (17, 30). Also, there were several differences in our study design compared to those of previous vesicle studies in marine plankton (16, 29) that may partially account for these results. These differences include our use of a 0.1 μm prefilter (compared to the 0.2 μm prefilters used in previous studies) and our use of CsCl density gradients, compared to the iodixanol gradients used in previous reports (16, 29). Consistent with previous viral metagenomic studies from the North Pacific Subtropical Gyre (26, 28), the virus fraction was dominated by viruses related to those that infect *Prochlorococcus* and *Pelagibacter* (see Fig. S3 in the supplemental material).

Our results reveal the first metagenomic characterization of free DNA, alongside two other exocellular DNA pools, vesicles and viruses. The importance of further characterizing the free DNA fraction is compounded by recent discoveries of the other exocellular DNA pools (viruses and vesicles) as potential vectors of genetic exchange in the marine environment (18) and cyanobacterial pili capable of utilizing exocellular DNA (31, 32).

Exocellular microbially derived DNA is not unique to the open ocean (3, 5, 21, 22). The terminology may vary, but free DNA has also been reported in terrestrial soils (33) and marine sediments (34), contributing up to 90% of the total DNA pool in marine sediments (35). Vibrio cholerae is known to take up exocellular DNA from dead cells, which is thought to shape antibiotic resistance, surface colonization, and intercellular communication (36). Cell-free DNA has also been documented in the human bloodstream; in some cases, it originates from tumor cells and can be used as a noninvasive method for cancer diagnosis (37). Free nucleic acids in the form of viroids (RNA) are even capable of causing infection in higher plants (38). Across microbial systems, a variety of functions have been attributed to cell-free DNA, exemplifying the vast evolutionary and ecological potential that free DNA may have in the open ocean.

In the open ocean, free DNA accounts for 25 to 50% of the total exocellular DNA (19, 21, 22) and has been shown to be rapidly consumed by microorganisms (12, 14, 21, 39). Whether it is used primarily as a nutrient source or for genetic exchange remains largely unknown. Previous work comparing the turnover of exocellular DNA pools indicates that free DNA is turned over more quickly than DNA inside viruses (21, 40), suggesting that it may be more readily available than structurally enclosed exocellular DNA (i.e., viruses and vesicles). Our investigation into free DNA reveals that there are different microbial sources for this material at the surface (75 to 125 m) than in the mesopelagic zone (250 to 1,000 m), implicating distinct ecological and evolutionary consequences of free DNA throughout the water column.

At the surface (75 to 125 m), free DNA sequences were dominated by viral and bacterial sequences, potentially reflecting active viral lysis at these depths. Previous metagenomic work at Station ALOHA revealed that predicted viral protein markers for lysogeny (integrase, CI repressor, and excisionase) were largely absent in near-surface waters and increased in prevalence below the deep chlorophyll maximum (28). At depth, cell abundances and primary production levels are lower than those at the surface (8), providing less energy and fewer hosts for viral replication (26, 41, 42). This depth-dependent variability in viral replication strategies at Station ALOHA coincides with the marked decrease in virus-derived free DNA sequences in the mesopelagic zone, the same depths at which fewer free phage sequences (0.02 μm filtered) (28) and enumerated free phage (21) are observed. Conversely, when cells are lysed, their intracellular contents are lost to the surrounding seawater. Indeed, viral lysis has been documented as a mechanism for the production of both total exocellular DNA (43) and free DNA (21, 40). Furthermore, an experiment documenting the production of free ribosomes following viral lysis of *Synechococcus* supports the possibility that viral lysis may be an important process introducing nucleic acids into the marine environment (44). Interrupted phage packaging has not yet been explicitly described in marine systems, but there is evidence for unpackaged DNA from nuclease treatments following cyanophage infection of dominant picocyanobacteria (45, 46) as well as in other microbial systems (47, 48). Together, our results suggest that viral lysis of bacterial cells at the surface may be important for the production of free DNA in the open ocean. Protozoan grazing (12, 43) and cell exudation (39, 49) have also been shown to result in increased exocellular DNA and might play significant roles in the production of free DNA in the open ocean. More research, separating the exocellular DNA pools is needed to uncover the source and dynamics behind these production mechanisms.

The high proportion of presumptive surface water-derived DNA sequences found in mesopelagic exocellular DNA suggests the potential downward export of either cells or free DNA as a delivery mechanism (Fig. 2). The exact mechanisms of free DNA delivery to the mesopelagic zone have yet to be documented. Possible explanations can be inferred from previous studies, including sediment trap and water column metagenomic analyses (25, 50), flux calculations of sinking particle disaggregation and degradation (51, 52), and migratory zooplankton (53). All of these mechanisms begin by defining the source of free DNA, which presumably originates from autochthonous particulate DNA (including cellular DNA, detrital DNA, and environmental DNA), reflecting the microbial communities present at the collection depth, or from allochthonous particulate DNA. Recently, surface-originated particulate DNA (as filtered cells) has been documented in the mesopelagic zone. For example, *Prochlorococcus* sequences have been recovered from sediment traps deployed at Station ALOHA (50), and their presence has been hypothesized to be the result of adsorption onto particles (and/or colloids) or from fecal pellets. Additionally, a recent investigation of core microbiome populations at Station ALOHA consistently found high-light *Prochlorococcus* ecotypes originating from the upper euphotic zone, at 500 to 1,000 m, in the particulate DNA at Station ALOHA (25). Consistent with this observation, our work demonstrates that high-light *Prochlorococcus* also dominates the free DNA at similar depths (Table S3), implicating surface water export.

With this evidence of particulate DNA exported from the surface, rapid surface turnover of free DNA, and mobilization by migratory zooplankton, it is assumed that the majority of DNA leaves the surface in the particulate phase rather than being dissolved (exocellular). Following the sinking of particulate DNA, it is hypothesized that particles disaggregate biologically and/or mechanically (52), and remineralization occurs by free-living or suspended microorganisms in the water column rather than microbial decomposition of sinking particles (54). This conceptual view stems from studies investigating carbon flux, nutrient contents of particles, on-particle metabolic activity, and accompanying models. Measurements of biomass production (particulate ATP) demonstrated that there was a net loss of living material on particles as a function of depth (54). From this and the accompanying rate measurements, it was concluded that sinking particles were not likely to be sites of active microbial decomposition. Similarly, Collins et al. (52) compared the on-particle metabolic activity to the metabolic demand of the water column itself. They found that the measurement of on-particle remineralization could not account for particle flux attenuation, suggesting that microbial decomposition occurs in the water column rather than on sinking particles.

By estimating the flux of particulate DNA out of the euphotic zone, delivery of DNA to the mesopelagic zone (by way of sinking particles) can be estimated (see Equation S1 in the supplemental material). This calculation assumes that (i) particulate DNA becomes free DNA by sinking particle disaggregation rather than on-particle degradation (52), (ii) the flux of DNA conforms to a Martin curve (55), (iii) DNA adheres to export efficiency and flux attenuation values determined from decades of production and export analyses at Station ALOHA (56), and (iv) an ecosystem steady state that is time invariant exists. This calculation predicts that the daily flux of DNA at 500 m is approximately 12 to 24% of the concentrations of free DNA reported at 500 m and 3 to 8% of those at 1,000 m (Equation S1) (21, 22). This calculation is consistent with our finding that 19% and 10% of the 500 m and 1,000 m annotated free DNA sequences were derived from surface-originated *Prochlorococcus*. Despite their small size, picophytoplankton like *Prochlorococcus* that dominate primary production in oligotrophic oceans are theorized to contribute proportional carbon export values (51). This suggests that carbon exported from the surface ocean is likely to be directly or indirectly derived from *Prochlorococcus* and other primary producers; therefore, surface-derived sequences are likely to contribute significantly to mesopelagic zone free DNA.

However, analyses revealed that free DNA from 500 and 1,000 m had more surface-derived sequences than the Martin curve and field data (57, 58) predict, 67 and 46% of euphotic zone-derived (0 to 200 m) sequences, respectively. If we assume that gene annotations accurately reflect exocellular DNA depth origins, a significant fraction of surface DNA remains unaccounted for, solely by sinking particles, suggesting that other mechanisms may supplement free DNA delivery to the mesopelagic zone. Delivery and free DNA standing stocks may also depend on depth-dependent turnover rates of free DNA, which are still not well constrained. Another important consideration is water mass controls on the supply of surface free DNA. Based on decades of salinity and temperature measurements at Station ALOHA (59), the water column is comprised of distinct water masses, including the North Pacific Intermediate Water, which manifests at Station ALOHA at around 500 to 770 m (60). This water mass is estimated to have been in contact with the atmosphere only 30 to 60 years ago according to a study (61) that used transient tracers to estimate the apparent water mass age at Station ALOHA. This provides evidence for other additional mechanisms of delivery of surface DNA to the mesopelagic zone.

Other possible mechanisms for delivering surface DNA to the mesopelagic zone may be more episodic in nature, for example, the passage and disaggregation of organic matter like the summer export pulse (62, 63), migratory zooplankton (53), and aggregates formed by picophytoplankton transparent exopolymer particles (64) and/or clay-ballasted particles (65). Recent analyses showed that sinking particulate organic matter was relatively energy replete, suggesting that a portion of deep organic carbon may be surface derived (66). Investigations of the mechanisms delivering free DNA and other labile dissolved organic matter (DOM) constituents are critical for expanding our understanding of water column dynamics and the biological carbon pump.

In total, the results highlight the potential ecological contributions of exocellular DNA with respect to the cycling of limiting nutrients (nitrogen and phosphorus) in the open ocean as well as a viable vector of genetic exchange. While these results have important implications, more research into the dynamics of free DNA is required to understand how this molecule is transformed from the particulate phase, its connection to microbially mediated food webs, and its capacity to carry surface microbial genes to the mesopelagic zone.

## MATERIALS AND METHODS

### Sample collection.

Seawater samples were collected during three cruises near Station ALOHA (22°45′N, 158°00′W), *R/V Kilo Moana* cruise HOT-297 (November 2017), *R/V Falkor* cruise FK180310 (April 2018), and *R/V Ka’imikai-O-Kanaloa* cruise HOT-302 (May 2018), using standard Niskin-type bottles set in a rosette water sampling system equipped with conductivity, temperature, depth (CTD) sensors. Sampling and analytical protocols for environmental measurements (inorganic nutrients [nitrate plus nitrite and phosphate], chlorophyll, temperature, salinity, *Prochlorococcus*, and heterotrophic bacterial cell abundances) were identical to those used by the Hawaii Ocean Time-series (HOT) program (http://hahana.soest.hawaii.edu/index.html). Twenty-liter exocellular DNA samples (vesicles, viruses, and free DNA) were collected and separated according to methods described previously by Linney et al. (22). In brief, this method includes three primary steps: (i) prefiltration directly from the Niskin-type bottles through a double-layered 0.1 μm polyethersulfone (PES) capsule filter (Acropak 1,000 cm^2^; Pall), (ii) concentration by tangential flow ultrafiltration (Pellicon 2, 0.1 m^2^ Ultracel membrane, 30 kDa nominal molecular weight limit [NMWL], >50 bp DNA; Millipore) down to ∼1 mL, and (iii) exocellular DNA separation by density gradient ultracentrifugation in cesium chloride. Following separation, exocellular DNA constituents are buffer exchanged with either TE (10 mM Tris-HCl, 1 mM disodium EDTA [pH 7.5]) (vesicles and free DNA) or SM (100 mM sodium chloride, 8 mM magnesium sulfate, 50 mM Tris-HCl [pH 7.5]) (viruses) buffer. This method ensures that all exocellular DNA (>50 bp) constituents are collected from the same sample, enabling comparison.

In the development of this method (22), a number of confirmation assays were employed to assess the application of this method; these include transmission electron microscopy, DNase treatment, macromolecular (proteins, RNA, and DNA) quantification, epifluorescence detection of virus-like particles, and fragment length analyses. These assays revealed that this method was able to directly separate vesicles, viruses, and free DNA collected from Station ALOHA while recovering large quantities of exocellular DNA (65 to 80%) and maintaining the integrity of the samples (i.e., no shearing or degradation of free DNA). Additionally, the prefiltration procedure was tested to determine whether microbial cells may break during this process and artifactually produce free DNA (22). Free DNA was not found to be produced by the prefiltration method, indicating its viability for separating cells from exocellular DNA. These confirmation assays, along with the ability to directly collect all exocellular DNA fractions from the same sample, supported the application of this method for determining the sources contributing exocellular DNA in the North Pacific Subtropical Gyre.

### Molecular weight determination of exocellular free DNA.

The size distributions of isolated free DNA were analyzed by capillary electrophoresis (Fragment Analyzer automated CE system; Advanced Analytical Technologies, Inc.) with a 33-cm capillary using a high-sensitivity genomic DNA analysis kit. Samples were run according to the manufacturer’s instructions (protocol DNF-488-33; Agilent). Digital sample peaks (electropherograms) were generated using ProSize 2.0 software.

### DNA extraction and purification.

DNA was extracted from viruses, vesicles, and free DNA (100 μL each sample) by first lysing and digesting samples in sucrose lysis buffer (final concentrations of 40 mM EDTA, 50 mM Tris [pH 8.3], and 0.75 M sucrose plus lysozyme [final concentration of 0.5 mg mL^−1^]) at 37°C for 30 min. Proteinase K (final concentration of 0.8 mg mL^−1^) and SDS (final concentration of 0.8%) were added, and the lysate was further incubated at 55°C for 2 h. The DNA was purified using a Chemagen MSM I instrument with the saliva DNA CMG-1037 kit (Perkin-Elmer, Waltham, MA). The quantity and quality of exocellular DNA samples and sequencing libraries were assessed by PicoGreen double-stranded DNA (dsDNA) quantitation (Invitrogen, Waltham, MA) and by capillary electrophoresis using a Fragment Analyzer automated CE system (protocol DNF-488-33).

### Metagenomic library preparation.

Exocellular DNA (vesicles, viruses, or free DNA) metagenomic libraries were prepared using a robotic liquid handling protocol (epMotion, Eppendorf, Hamburg, DE) with a TruSeq Nano DNA library preparation kit (catalog number 15041110; Illumina); a DNA input per sample of 2 ng μL^−1^ sheared to an average size of 350 bp utilizing a Covaris M220 focused ultrasonicator according to the manufacturer’s recommendations, with modifications to the shear time to target 350 bp; and Microtube-50 AFA fiber tubes. (For some selected samples that had bimodal DNA size distributions, two sequencing libraries were prepared, one with DNA shearing and one without DNA shearing.) Metagenome sequencing libraries were prepared using Illumina’s TruSeq Nano LT library preparation kit and sequenced on an Illumina Nextseq500 system, using the V2 high-output 300-cycle reagent kit, with the addition of 1% of a PhiX control.

### DNA sequence analysis and annotation.

Sequenced exocellular DNA reads were filtered and trimmed for quality using the iu-filter-quality-minoche tool (67) from illumina-utils (68). Quality-filtered reads were identified and counted by mapping against the ALOHA 2.0 metagenomic reference gene catalog (7, 26) using lastal (69). Reads mapping against the ALOHA 2.0 gene catalog with 90% identity or better over at least 25 amino acids (AAI [amino acid identity]) were collected into a gene count table for each exocellular DNA data set.

Gene counts in all samples were normalized by the total number of mapped genes. The family-level taxonomic abundance was measured by the number of gene counts assigned to each microbial family. The relative abundances of quality-controlled exocellular DNA gene counts were used to assess the variation among exocellular DNA sample types (vesicle, virus, and free DNA) and across the water column (75 to 1,000 m). The most abundant (“major”) bacterial and viral families were determined by a contribution of >0.5% (cutoff) to all exocellular DNA samples and were compiled into proportional gene count tables. These assessments revealed the microbial sources of the exocellular DNA samples as well as the known viral hosts and *Prochlorococcus* ecotype proportions.

To estimate the proportions of exocellular DNA sequences from each metagenomic sample that may have originated from a particular water column depth (0 to 4,000 m), we compared the normalized coverage that the ALOHA 2.0 catalog (7, 26) had at each depth in the ALOHA 2.0 survey to the normalized coverage of each gene in our samples. First, we calculated the probability that a sequence matching a given gene originated at a given depth as the ratio of the coverage of that gene at that depth to the total coverage of the gene. Next, for all depths, we multiplied that probability by the normalized coverage for each gene in our metagenomic exocellular DNA samples to obtain the portion of that coverage that likely originated at each depth. The coverages, now portioned by probable depth origin, were then aggregated by domain (bacterial, archaeal, eukaryote, viral, or unknown) to better understand potential source patterns.

### Comparison to cellular and viral data sets collected from Station ALOHA.

All exocellular DNA gene count tables (vesicles, viruses, and free DNA) were combined and mapped to previously reported cellular (7) and viral (26) ALOHA 2.0 metagenomic reference gene catalogs collected on multiple HOT research cruises from the same depths at Station ALOHA (75, 100, 125, 250, 500, and 1,000 m). Multivariate pairwise comparisons (PERMANOVA) were analyzed using the community ecology package in R (adonis function, vegan package) (70) to compare exocellular DNA with cellular and viral Station ALOHA samples. Read counts per taxonomic clade were normalized by calculating their proportions relative to the total number of mapped genes per sample. Normalized values were compiled in a proportional order-level count table and square root transformed. Two-dimensional ordination methods were used on normalized count tables to compare exocellular DNA data sets to cellular and viral samples from the same depths. To visualize distances, nonmetric multidimensional scaling (NMDS) plots were generated using the metaMDS function and Bray-Curtis distance matrices constructed from normalized gene counts in the vegan R package.

### Data availability.

Genomic data and raw reads are available under NCBI BioProject accession number PRJNA727670.

## References

[1] Holm-Hansen O, Sutcliffe WH, Jr, Sharp J. 1968. Measurement of deoxyribonucleic acid in the ocean and its ecological significance. Limnol Oceanogr 13:507–514. 10.4319/lo.1968.13.3.0507.

[2] Winn CD, Karl DM. 1986. Diel nucleic acid synthesis and particulate DNA concentrations: conflicts with division rate estimates by DNA accumulation. Limnol Oceanogr 31:637–645. 10.4319/lo.1986.31.3.0637.

[3] DeFlaun MF, Paul JH, Jeffrey WH. 1987. Distribution and molecular weight of dissolved DNA in subtropical estuarine and oceanic environments. Mar Ecol Prog Ser 38:65–73. 10.3354/meps038065.

[4] Dell’Anno A, Marrale D, Pusceddu A, Fabiano M, Danovaro R. 1999. Particulate nucleic acid dynamics in a highly oligotrophic system: the Cretan Sea (Eastern Mediterranean). Mar Ecol Prog Ser 186:19–30. 10.3354/meps186019.

[5] Karl DM, Bailiff MD. 1989. The measurement and distribution of dissolved nucleic acids in aquatic environments. Limnol Oceanogr 34:543–558. 10.4319/lo.1989.34.3.0543.

[6] Djurhuus A, Closek CJ, Kelly RP, Pitz KJ, Michisaki RP, Starks HA, Walz KR, Andruszkiewicz EA, Olesin E, Hubbard K, Montes E, Otis D, Muller-Karger FE, Chavez FP, Boehm AB, Breitbart M. 2020. Environmental DNA reveals seasonal shifts and spatial interactions in a marine community. Nat Commun 11:254. 10.1038/s41467-019-14105-1. 31937756 PMC6959347

[7] Mende DR, Bryant JA, Aylward FO, Eppley JM, Nielsen T, Karl DM, DeLong EF. 2017. Environmental drivers of a microbial genomic transition zone in the ocean’s interior. Nat Microbiol 2:1367–1373. 10.1038/s41564-017-0008-3. 28808230

[8] Karl DM, Church MJ. 2014. Microbial oceanography and the Hawaii Ocean Time-series programme. Nat Rev Microbiol 12:699–713. 10.1038/nrmicro3333. 25157695

[9] Frischer ME, Thurmond JM, Paul JH. 1990. Natural plasmid transformation in a high-frequency-of-transformation marine *Vibrio* strain. Appl Environ Microbiol 56:3439–3444. 10.1128/aem.56.11.3439-3444.1990. 2268155 PMC184975

[10] Jeffrey WH, Paul JH, Stewart GJ. 1990. Natural transformation of a marine *Vibrio* species by plasmid DNA. Microb Ecol 19:259–268. 10.1007/BF02017170. 24196363

[11] Paul JH, Jiang SC, Rose JB. 1991. Concentration of viruses and dissolved DNA from aquatic environments by vortex flow filtration. Appl Environ Microbiol 57:2197–2204. 10.1128/AEM.57.8.2197-2204.1991. 1768090 PMC183550

[12] Turk V, Rehnstam A-S, Lundberg E, Hagström Å. 1992. Release of bacterial DNA by marine nanoflagellates, an intermediate step in phosphorus regeneration. Appl Environ Microbiol 58:3744–3750. 10.1128/aem.58.11.3744-3750.1992. 16348813 PMC183168

[13] Jørgensen NOG, Jacobsen CS. 1996. Bacterial uptake and utilization of dissolved DNA. Aquat Microb Ecol 11:263–270. 10.3354/ame011263.

[14] Lennon JT. 2007. Diversity and metabolism of marine bacteria cultivated on dissolved DNA. Appl Environ Microbiol 73:2799–2805. 10.1128/AEM.02674-06. 17337557 PMC1892854

[15] Hermansson M, Linberg C. 1994. Gene transfer in the marine environment. FEMS Microbiol Ecol 15:47–54. 10.1111/j.1574-6941.1994.tb00228.x.

[16] Biller SJ, Schubotz F, Roggensack SE, Thompson AW, Summons RE, Chisholm SW. 2014. Bacterial vesicles in marine ecosystems. Science 343:183–186. 10.1126/science.1243457. 24408433

[17] Morris RM, Cain KR, Hvorecny KL, Kollman JM. 2020. Lysogenic host-virus interactions in SAR11 marine bacteria. Nat Microbiol 5:1011–1015. 10.1038/s41564-020-0725-x. 32424337 PMC7387148

[18] Hackl T, Laurenceau R, Ankenbrand MJ, Bliem C, Cariani Z, Thomas E, Dooley KD, Arellano AA, Hogle SL, Berube P, Leventhal GE, Luo E, Eppley J, Zayed AA, Beaulaurier J, Stepanauskas R, Sullivan MD, DeLong EF, Biller SJ, Chisholm SW. 2020. Novel integrative elements and genomic plasticity in ocean ecosystems. bioRxiv. 10.1101/2020.12.28.424599. 36608657

[19] Brum JR, Steward GF, Karl DM. 2004. A novel method for the measurement of dissolved deoxyribonucleic acid in seawater. Limnol Oceanogr Methods 2:248–255. 10.4319/lom.2004.2.248.

[20] DeFlaun MF, Paul JH, Davis D. 1986. Simplified method for dissolved DNA determination in aquatic environment. Appl Environ Microbiol 52:654–659. 10.1128/aem.52.4.654-659.1986. 16347160 PMC239092

[21] Brum JR. 2005. Concentration, production and turnover of viruses and dissolved DNA pools at Stn ALOHA, North Pacific Subtropical Gyre. Aquat Microb Ecol 41:103–113. 10.3354/ame041103.

[22] Linney MD, Schvarcz CR, Steward GF, DeLong EF, Karl DM. 2021. A method for characterizing dissolved DNA and its application to the North Pacific Subtropical Gyre. Limnol Oceanogr Methods 19:210–221. 10.1002/lom3.10415.

[23] DeLong EF, Preston CM, Mincer T, Rich V, Hallam SJ, Frigaard N-U, Martinez A, Sullivan MB, Edwards R, Brito BR, Chisholm SW, Karl DM. 2006. Community genomics among stratified microbial assemblages in the ocean’s interior. Science 311:496–503. 10.1126/science.1120250. 16439655

[24] Brum JR, Culley AI, Steward GF. 2013. Assembly of a marine viral metagenome after physical fractionation. PLoS One 8:e60604. 10.1371/journal.pone.0060604. 23580170 PMC3620275

[25] Mende DR, Boeuf D, DeLong EF. 2019. Persistent core populations shape the microbiome throughout the water column in the North Pacific Subtropical Gyre. Front Microbiol 10:2273. 10.3389/fmicb.2019.02273. 31632377 PMC6779783

[26] Luo E, Eppley JM, Romano AE, Mende DR, DeLong EF. 2020. Double-stranded DNA virioplankton dynamics and reproductive strategies in the oligotrophic open ocean water column. ISME J 14:1304–1315. 10.1038/s41396-020-0604-8. 32060418 PMC7174320

[27] Aylward FO, Boeuf D, Mende DR, Wood-Charlson EM, Vislova A, Eppley JM, Romano AE, DeLong EF. 2017. Diel cycling and long-term persistence of viruses in the ocean’s euphotic zone. Proc Natl Acad Sci USA 114:11446–11451. 10.1073/pnas.1714821114. 29073070 PMC5663388

[28] Luo E, Aylward FO, Mende DR, DeLong EF. 2017. Bacteriophage distributions and temporal variability in the ocean’s interior. mBio 8:e01903-17. 10.1128/mBio.01903-17. 29184020 PMC5705922

[29] Biller SJ, McDaniel LD, Breitbart M, Rogers E, Paul JH, Chisholm SW. 2017. Membrane vesicles in sea water: heterogenous DNA content and implications for viral abundance estimates. ISME J 11:394–404. 10.1038/ismej.2016.134. 27824343 PMC5270575

[30] Zhao X, Schwartz CL, Pierson J, Giovannoni SJ, McIntosh JR, Nicastro D. 2017. Three-dimensional structure of the ultraoligotrophic marine bacterium “*Candidatus* Pelagibacter ubique.” Appl Environ Microbiol 83:e02807-16. 10.1128/AEM.02807-16. 27836840 PMC5244296

[31] Taton A, Erikson C, Yang Y, Rubin BE, Rifkin SA, Golden JW, Golden SS. 2020. The circadian clock and darkness control natural competence in cyanobacteria. Nat Commun 11:1688. 10.1038/s41467-020-15384-9. 32245943 PMC7125226

[32] Aguilo-Ferretjans MDM, Bosch R, Puxty RJ, Latva M, Zadjelovic V, Chhun A, Sousoni D, Polin M, Scanlan DJ, Christie-Oleza JA. 2021. Pili allow dominant marine cyanobacteria to avoid sinking and evade predation. Nat Commun 12:1857. 10.1038/s41467-021-22152-w. 33767153 PMC7994388

[33] Pietramellara G, Ascher J, Borgogni F, Ceccherini MT, Guerri G, Nannipieri P. 2009. Extracellular DNA in soil and sediment: fate and ecological relevance. Biol Fertil Soils 45:219–235. 10.1007/s00374-008-0345-8.

[34] Torti A, Lever MA, Jørgensen BB. 2015. Origin, dynamics, and implications of extracellular DNA pools in marine sediments. Mar Genomics 24:185–196. 10.1016/j.margen.2015.08.007. 26452301

[35] Dell’Anno A, Stefano B, Danovaro R. 2002. Quantification, base composition, and fate of extracellular DNA in marine sediments. Limnol Oceanogr 47:899–905. 10.4319/lo.2002.47.3.0899.

[36] Ellison CK, Dalia TN, Vidal Ceballos A, Wang JC-Y, Biais N, Brun YV, Dalia AB. 2018. Retraction of DNA-bound type IV competence pili initiates DNA uptake during natural transformation in *Vibrio cholerae*. Nat Microbiol 3:773–780. 10.1038/s41564-018-0174-y. 29891864 PMC6582970

[37] Stewart CM, Tsui DWY. 2018. Circulating cell-free DNA for non-invasive cancer management. Cancer Genet 228–229:169–179. 10.1016/j.cancergen.2018.02.005. PMC6598437 29625863

[38] Adkar-Purushothama CR, Perreault J-P. 2020. Current overview on viroid-host interactions. Wiley Interdiscip Rev RNA 11:e1570. 10.1002/wrna.1570. 31642206

[39] Paul JH, Jeffrey WH, DeFlaun MF. 1987. Dynamics of extracellular DNA in the marine environment. Appl Environ Microbiol 53:170–179. 10.1128/aem.53.1.170-179.1987. 3827244 PMC203621

[40] Riemann L, Holmfeldt K, Titelman J. 2009. Importance of viral lysis and dissolved DNA for bacterioplankton activity in a P-limited estuary, Northern Baltic Sea. Microb Ecol 57:286–294. 10.1007/s00248-008-9429-0. 18670729

[41] Moebus K. 1996. Marine bacteriophage reproduction under nutrient-limited growth of host bacteria. I. Investigations with six phage-host systems. Mar Ecol Prog Ser 144:1–12. 10.3354/meps144001.

[42] Weinbauer MG, Brettar I, Höfle MG. 2003. Lysogeny and virus-induced mortality of bacterioplankton in surface, deep, and anoxic marine waters. Limnol Oceanogr 48:1457–1465. 10.4319/lo.2003.48.4.1457.

[43] Alonso MC, Rodriguez V, Rodriguez J, Borrego JJ. 2000. Role of ciliates, flagellates and bacteriophages on the mortality of marine bacteria and on dissolved-DNA concentration in laboratory experimental systems. J Exp Mar Biol Ecol 244:239–252. 10.1016/S0022-0981(99)00143-4.

[44] Zhong X, Wirth J, Suttle C. 2016. Ribosomes in the sea: a window on taxon-specific lysis, abstr MM44D-0513. Abstr Am Geophys Union Ocean Sci Meet 2016, 2016AGUOSMM44D0513S.

[45] Baran N, Goldin S, Maidanik I, Lindell D. 2018. Quantification of diverse virus populations in the environment using the polony method. Nat Microbiol 3:62–72. 10.1038/s41564-017-0045-y. 29085077 PMC5739286

[46] Zborowsky S, Lindell D. 2019. Resistance in marine cyanobacteria differs against specialist and generalist cyanophages. Proc Natl Acad Sci USA 116:16899–16908. 10.1073/pnas.1906897116. 31383764 PMC6708340

[47] Liljemark WF, Anderson DL. 1970. Morphology and physiology of the intracellular development of *Bacillus subtilis* bacteriophage ϕ25. J Virol 6:114–124. 10.1128/JVI.6.1.114-124.1970. 4990531 PMC376097

[48] Powell IB, Tulloch DL, Hillier AJ, Davidson BE. 1992. Phage DNA synthesis and host DNA degradation in the life cycle of *Lactococcus lactis* bacteriophage c6A. J Gen Microbiol 138:945–950. 10.1099/00221287-138-5-945. 1645131

[49] Paul JH, Jeffrey WH, Cannon JP. 1990. Production of dissolved DNA, RNA, and protein by microbial populations in a Florida reservoir. Appl Environ Microbiol 56:2957–2962. 10.1128/aem.56.10.2957-2962.1990. 1704697 PMC184883

[50] Fontanez KM, Eppley JM, Samo TJ, Karl DM, DeLong EF. 2015. Microbial community structure and function on sinking particles in the North Pacific Subtropical Gyre. Front Microbiol 6:469. 10.3389/fmicb.2015.00469. 26042105 PMC4436931

[51] Richardson TL, Jackson GA. 2007. Small phytoplankton and carbon export from the surface ocean. Science 315:838–840. 10.1126/science.1133471. 17289995

[52] Collins JR, Edwards BR, Thamatrakoln K, Ossolinski JE, DiTullio GR, Bidle KD, Doney SC, Van Mooy BAS. 2015. The multiple fates of sinking particles in the North Atlantic Ocean. Global Biogeochem Cycles 29:1471–1494. 10.1002/2014GB005037.

[53] Hannides CCS, Popp BN, Choy CA, Drazen JC. 2013. Midwater zooplankton and suspended particle dynamics in the North Pacific Subtropical Gyre: a stable isotope perspective. Limnol Oceanogr 58:1931–1946. 10.4319/lo.2013.58.6.1931.

[54] Karl DM, Knauer GA, Martin JH. 1988. Downward flux of particulate organic matter in the ocean: a particle decomposition paradox. Nature 332:438–441. 10.1038/332438a0.

[55] Martin JH, Knauer GA, Karl DM, Broenkow WW. 1987. VERTEX: carbon cycling in the northeast Pacific. Deep Sea Res A 34:267–285. 10.1016/0198-0149(87)90086-0.

[56] Karl DM, Letelier RM, Bidigare RR, Björkman KM, Church MJ, Dore JE, White AE. 2021. Seasonal-to-decadal scale variability in primary production and particulate matter export at Station ALOHA. Prog Oceanogr 195:102563. 10.1016/j.pocean.2021.102563.

[57] Karl DM, Christian JR, Dore JE, Hebel DV, Letelier RM, Tupas LM, Winn CD. 1996. Season and interannual variability in primary production and particle flux at Station ALOHA. Deep Sea Res 2 Top Stud Oceanogr 43:539–568. 10.1016/0967-0645(96)00002-1.

[58] Hebel DV, Karl DM. 2001. Seasonal, interannual and decadal variations in particulate matter concentrations and composition in the subtropical North Pacific Ocean. Deep Sea Res 2 Top Stud Oceanogr 48:1669–1695. 10.1016/S0967-0645(00)00155-7.

[59] Lukas R, Santiago-Mandujano F. 2008. Interannual to interdecadal salinity variations observed near Hawaii: local and remote forcing by surface freshwater fluxes. Oceanography 21:46–55. 10.5670/oceanog.2008.66.

[60] Talley LD. 1993. Distribution and formation of North Pacific Intermediate Water. J Phys Oceanogr 23:517–537. 10.1175/1520-0485(1993)023<0517:DAFONP>2.0.CO;2.

[61] Bullister JL, Wisegarver DP, Sonnerup RE. 2006. Sulfur hexafluoride as a transient tracer in the North Pacific Ocean. Geophys Res Lett 33:L18603. 10.1029/2006GL026514.

[62] Karl DM, Church MJ, Dore JE, Letelier RM, Mahaffey C. 2012. Predictable and efficient carbon sequestration in the North Pacific Ocean supported by symbiotic nitrogen fixation. Proc Natl Acad Sci USA 109:1842–1849. 10.1073/pnas.1120312109. 22308450 PMC3277559

[63] Poff KE, Leu AO, Eppley JM, Karl DM, DeLong EF. 2021. Microbial dynamics of elevated carbon flux in the open ocean’s abyss. Proc Natl Acad Sci USA 118:e2018269118. 10.1073/pnas.2018269118. 33479184 PMC7848738

[64] Cruz BN, Neuer S. 2019. Aggregation of the marine picocyanobacteria *Prochlorococcus* and *Synechococcus*. Front Microbiol 10:1864. 10.3389/fmicb.2019.01864. 31456778 PMC6700329

[65] Deng W, Monks L, Neuer S. 2015. Effects of clay minerals on the aggregation and subsequent settling of marine *Synechococcus*. Limnol Oceanogr 60:805–816. 10.1002/lno.10059.

[66] Grabowski E, Letelier RM, Laws EA, Karl DM. 2019. Coupling carbon and energy fluxes in the North Pacific Subtropical Gyre. Nat Commun 10:1895. 10.1038/s41467-019-09772-z. 31028256 PMC6486601

[67] Minoche AE, Dohm JC, Himmelbauer H. 2011. Evaluation of genomic high-throughput sequencing data generated on Illumina HiSeq and Genome Analyzer systems. Genome Biol 12:R112. 10.1186/gb-2011-12-11-r112. 22067484 PMC3334598

[68] Eren AM, Vineis JH, Morrison HG, Sogin ML. 2013. A filtering method to generate high quality short reads using Illumina paired-end technology. PLoS One 8:e66643. 10.1371/journal.pone.0066643. 23799126 PMC3684618

[69] Kiełbasa SM, Wan R, Sato K, Horton P, Frith MC. 2011. Adaptive seeds tame genomic sequence comparison. Genome Res 21:487–493. 10.1101/gr.113985.110. 21209072 PMC3044862

[70] Oksanen J, Blanchet FG, Friendly M, Kindt R, Legendre P, McGlinn D, Minchin PR, O’Hara RB, Simpson GL, Solymos P, Stevens MHH, Szoecs E, Wagner H. 2017. vegan: community ecology package. R package version 2.5-7. https://CRAN.R-project.org/package=vegan.

