# Supplementary material for: Microbial Sources of Exocellular DNA in the Ocean
Source: Appl Environ Microbiol. 2022 Mar 21;88(7):e02093-21. doi: 10.1128/aem.02093-21 (PMC9004351; doi:10.1128/aem.02093-21)
Supplement: Supplemental file 1 — Text S1, Tables S1 to S4, Fig. S1 to S4, Equations S1 to S4, and descriptions of Data Sets S1 to S4. Download aem.02093-21-s0001.pdf, PDF file, 2.8 MB [file aem.02093-21-s0001.pdf]

Supplementary Material

Title: Microbial sources of exocellular DNA in the ocean

Authors: Morgan D. Linney[1*], John M. Eppley[1], Anna E. Romano[1], Elaine Luo[1], Edward F. DeLong[1], David M. Karl[1]

Affiliations:
[1]Department of Oceanography, Daniel K. Inouye Center for Microbial Oceanography: Research and Education (C-MORE), University of Hawaiʻi at Mānoa, Honolulu, HI 96822, USA.

*Corresponding author. Email: linney@mit.edu

Document Contents:

1. Supplementary Text (Results)
2. Table S1: Cruise sampling summary table
3. Table S2: Total number of gene counts for each exocellular DNA sample (vesicles, viruses, and free DNA)
4. Table S3: Proportion of *Prochlorococcus* HL sequences present in each exocellular metagenomic DNA sample (vesicles, viruses, and free DNA)
5. Table S4. Microscopy and molecular characterizations of the cesium chloride gradient pools
6. Figure S1. Water mass structure, nutrients, and cell abundance depth profiles at Station ALOHA
7. Supplementary Equation
8. Figure S2. Representative micrographs from each exocellular DNA fraction.
9. Figure S3. Diversity of host-specified phages contributing to the three exocellular DNA pools.
10. Figure S4A-B. Bray-Curtis dissimilarity based non-metric multidimensional scaling (NMDS) of exocellular DNA fractions

Supplementary Spreadsheets:

1. DatasetS1: All family-level annotated taxon counts for all exocellular metagenomic DNA (vesicles, viruses, and free DNA) samples
2. DatasetS2: Major bacterial and viral families (cutoff: major family contributes ≥0.65% of all exocellular DNA samples) contributing to exocellular metagenomic DNA (vesicles, viruses, and free DNA) gene counts. Data used in main text (as proportions).
3. DatasetS3: Host-associated phage gene counts for each exocellular metagenomic DNA sample (vesicles, viruses, and free DNA) annotated from the ALOHA 2.0 catalog metadata
4. DatasetS4: Depth-assigned gene counts for each exocellular metagenomic DNA sample type (vesicles, viruses, and free DNA) annotated from the ALOHA 2.0 catalog metadata. Data used in main text (as proportions).

Supplementary Text (Results):

A. Confirmation of exocellular free DNA isolation

The method (1) applied to isolate vesicles, viruses, and free DNA was validated by a number of experimental tests. In brief, the method concentrates large volumes of prefiltered (0.1 μm) seawater by ultrafiltration (30 kDa), then separates exocellular DNA pools by density gradient ultracentrifugation. This allows for the direct examination of these three components. Following this separation these three pools were subjected to a number of experiments to assess the efficacy of this method. These experiments involved: visualization analyses (epifluorescence and transmission electron microscopy), macromolecular quantification (fluorescence quantification of proteins, RNA, and DNA), and DNase treatment. From these examinations it was determined that free DNA was present in the most dense (1.6-1.7 g mL^{-1} in cesium chloride) fraction of the density gradient. Visual examinations of this free DNA isolate, confirmed the presence of DNA and the absence of viruses or vesicles. There was also a near complete (92-97%) degradation of fluorescently quantified DNA signal in this isolate following DNase treatment (Table S3). The utilization of independent evaluation methods helped confirm its application for free DNA isolation, and warranted further investigations to identify its biological composition alongside the other exocellular DNA pools.

Table S1. Cruise sampling summary table

**Characteristic**	**Cruise 1: HOT297**	**Cruise 2: FK180310**	**Cruise 3: HOT302**
Sampling date	November 2017	April 2018	May 2018
Location	22°45’N, 158°00’W	22°17’N, 157°01’W	22°45’N, 158°00’W
Sample depths (m)	75, 125, 500	100, 250	1000
Deep chlorophyll maximum depth (m)	125	120	125

Table S2. Total number of gene counts for each exocellular DNA sample (vesicles, viruses, and free DNA).

Sample Name	Sample Collection Depth (m)	Exocellular DNA Type	Total read counts	Total annotated genes	Total family-level annotated gene counts	BioProject	Accession
Ves75	75	Vesicle	12747332	12255903	6657660	PRJNA727670	SRX10803060
Ves125	125	Vesicle	15397229	15166035	11550100	PRJNA727670	SRX10803069
Ves500	500	Vesicle	16694964	15979742	11152500	PRJNA727670	SRX10803072
Vir75	75	Virus	14362092	13970978	5420090	PRJNA727670	SRX10803070
Vir100	100	Virus	13363478	13080771	5031730	PRJNA727670	SRX10803058
Vir125	125	Virus	14031653	13693086	4942910	PRJNA727670	SRX10803075
Vir250	250	Virus	13206072	12815625	3645800	PRJNA727670	SRX10803057
Vir500	500	Virus	16163098	15551348	4223860	PRJNA727670	SRX10803073
FDNA75	75	Free DNA	12427745	11879884	6651250	PRJNA727670	SRX10803059
FDNA100	100	Free DNA	7737206	7585269	3862660	PRJNA727670	SRX10803061
FDNA125	125	Free DNA	14886720	11639558	6976200	PRJNA727670	SRX10803066
FDNA250	250	Free DNA	10650078	8509298	5337180	PRJNA727670	SRX10803062
FDNA500	500	Free DNA	14638768	13600011	9109520	PRJNA727670	SRX10803071
FDNA1000	1000	Free DNA	14653482	9800715	6138520	PRJNA727670	SRX10803068

Table S3. Proportion of *Prochlorococcus* HL sequences present in each exocellular metagenomic DNA sample (vesicles, viruses, and free DNA)

Sample Name	Sample Collection Depth (m)	Exocellular DNA Type	Sample Location	Cruise Number	% Prochlorococcus	% HL Pro (of Pro Proportion)
Ves75	75	Vesicle	(22° 45'N, 158° 00'W)	HOT297	0.956148566	89.71561549
Ves125	125	Vesicle	(22° 45'N, 158° 00'W)	HOT297	0.144640362	84.8414816
Ves500	500	Vesicle	(22° 45'N, 158° 00'W)	HOT297	0.235137718	96.39305541
Vir75	75	Virus	(22° 45'N, 158° 00'W)	HOT297	8.057730786	66.48090183
Vir100	100	Virus	(22° 17'N, 157° 01'W)	FK180310	5.190158289	64.86248999
Vir125	125	Virus	(22° 45'N, 158° 00'W)	HOT297	5.447230871	50.02645504
Vir250	250	Virus	(22° 17'N, 157° 01'W)	FK180310	6.142739664	95.19898841
Vir500	500	Virus	(22° 45'N, 158° 00'W)	HOT297	7.210657977	96.17837208
FDNA75	75	Free DNA	(22° 45'N, 158° 00'W)	HOT297	4.84542706	86.98343606
FDNA100	100	Free DNA	(22° 17'N, 157° 01'W)	FK180310	7.083801197	86.23890143
FDNA125	125	Free DNA	(22° 45'N, 158° 00'W)	HOT297	41.35865219	88.75259378
FDNA250	250	Free DNA	(22° 17'N, 157° 01'W)	FK180310	6.651181999	55.31197202
FDNA500	500	Free DNA	(22° 45'N, 158° 00'W)	HOT297	40.89583484	99.81282631
FDNA1000	1000	Free DNA	(22° 45'N, 158° 00'W)	HOT302	30.02829943	93.06218841

Table S4. Microscopy and molecular characterizations of the cesium chloride gradient pools. The recovery of DNA from each pool had a range of 68-86%.

Pool	Density in cesium chloride	Dominant contents (examined by TEM)	Epifluorescence VLP detection	% DNA remaining post-DNase Treatment	DNA:protein range
Vesicles	(1.30–1.35 g mL^{-1})	Round vesicles	VLP detected	89–100	1.2–2.3
Viruses	(1.40–1.55 g mL^{-1})	Viruses (various morphologies)	VLP detected	88–98	4.3–6.8
Free DNA	(1.60–1.70 g mL^{-1})	Linear structures (DNA)	VLP absent	3–8	30.6–42.1

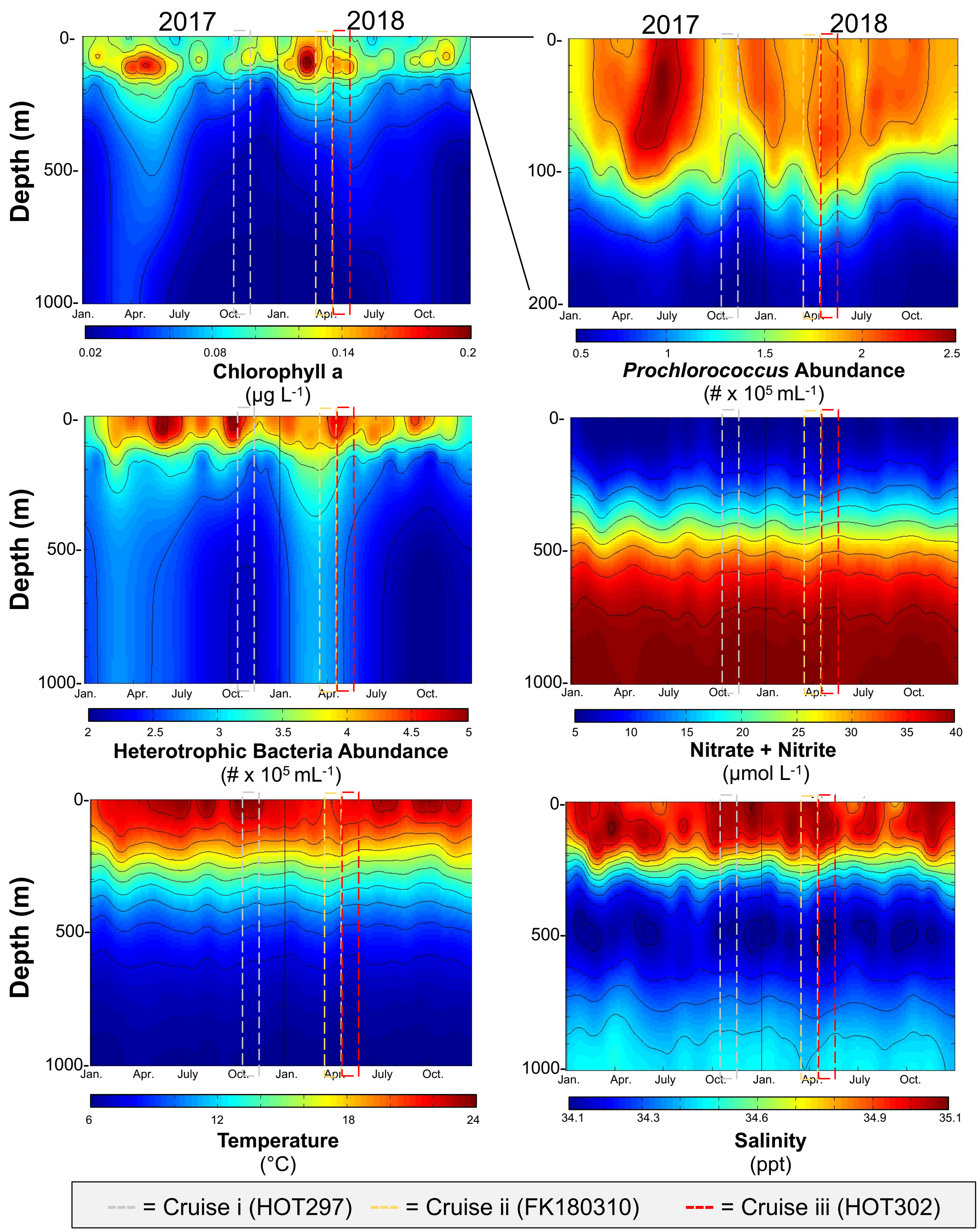

Figure S1. Nutrients and cell abundance depth profiles at Station ALOHA in November. Depth profiles of environmental data at Station ALOHA during the sampling period (2017-2018). (a) fluorometric chlorophyll (b) *Prochlorococcus* and (c) heterotrophic bacteria cell abundances (d) nitrate + nitrite (e) temperature, and (f) salinity

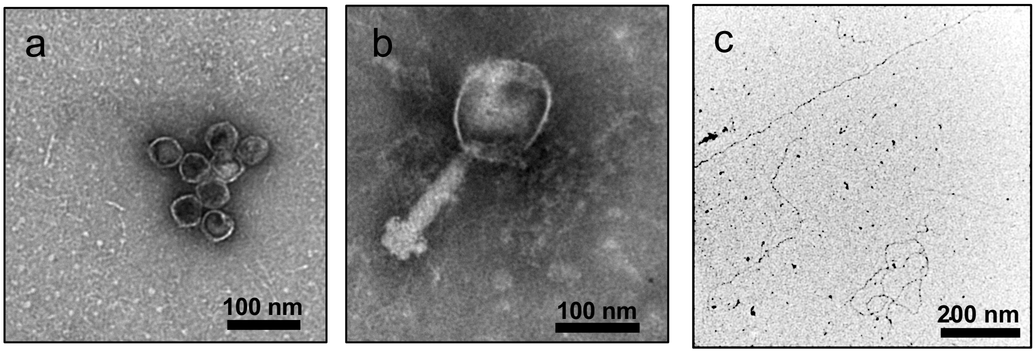

Figure S2. Representative micrographs from each exocellular DNA fraction. (a) vesicles isolated from the least dense fraction (1.30-1.35 g/mL in CsCl), (b) virus isolated from the mid-density fraction (1.40-1.55 g/mL in CsCl), (c) free DNA isolated from the most dense fraction (1.60-1.70 g/mL in CsCl). Total exocellular DNA concentrations ranged from 0.17-0.45 µg L^{-1} with the amount of DNA between these three accounting for <10% of the sum of all density fractions (<0.02µg L^{-1}).

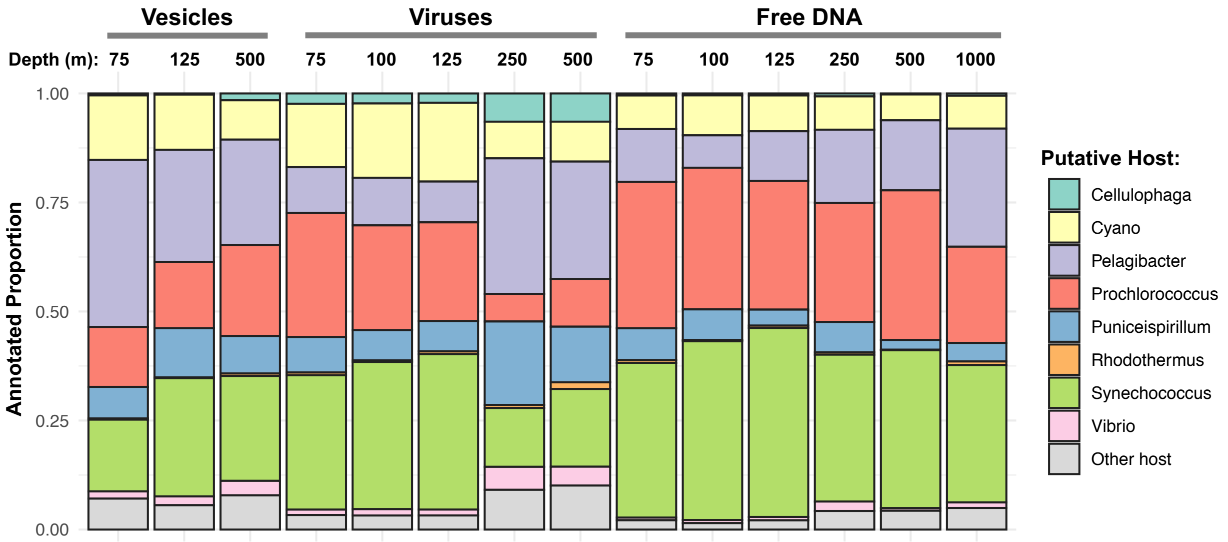

Figure S3. Diversity of host-specified phages contributing to the three exocellular DNA pools. Values are proportions of genes derived from viruses annotated to known hosts. The dominant phage hosts were determined by a contribution of >0.05% (cutoff) to all exocellular metagenomic gene counts, resulting in eight associated hosts across the three exocellular DNA pools.

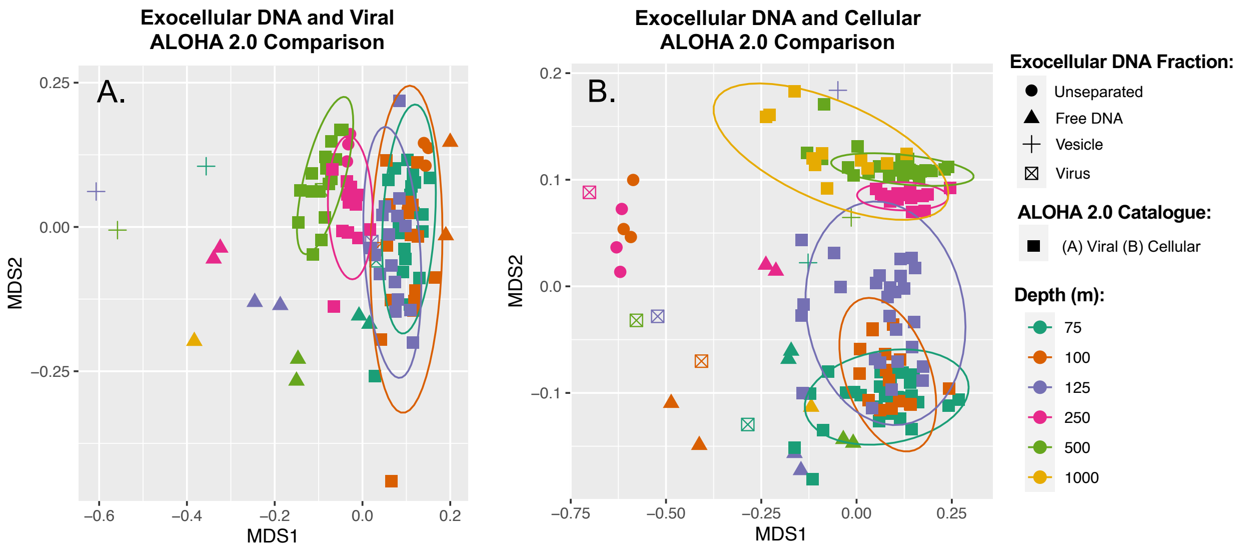

Figure S4. Comparison of exocellular DNA samples with cellular and viral datasets collected from Station ALOHA. Bray-Curtis dissimilarity based non-metric multidimensional scaling (NMDS) of exocellular DNA samples (vesicles, viruses, and free DNA) and ALOHA 2.0 gene catalog family-level annotated abundances. Ellipsoids represent 95% CI around the centroid. “Unseparated exocellular DNA” samples were samples collected by the same exocellular DNA ultrafiltration methods, but were not separated by density. (A) Comparison of viral ALOHA 2.0 gene catalog (squares) with all exocellular DNA fractions. Surface free DNA (75 and 100 m) cluster with viral catalog samples from the same depth, while deeper (125-1000 m) free DNA and vesicle (all depths) cluster separately from the viral catalog samples and to a lesser degree, each other. “Virus” exocellular DNA samples cluster with viral catalog samples from the same depths. (b) Analysis with cellular ALOHA 2.0 gene catalog and all exocellular DNA fractions. Virus-enriched exocellular DNA samples (“Virus”, “Unseparated exocellular DNA”, and free DNA from 100 m) cluster together, and separately from cellular ALOHA 2.0 samples. Deeper free DNA (500-1000 m) samples cluster with cellular ALOHA 2.0 datasets from the upper euphotic (75-100m) zone.

Supplementary Equation:

1. <u>Calculate flux (F_{eu}) of particulate DNA out of the euphotic zone:</u>
   F_{eu} = Net primary production (NPP) * export efficiency (ef) * P-DNA:PC
   NPP = 500 mgC m^{-2} day^{-1} (2)
   ef = 0.055 (2)
   P-DNA= 0.2 mg m^{-2} (Value at 100 m in North Pacific from 3)
   PC = 1.5 mg m^{-2} (Approximate value at 100 m acquired from HOTDOGs)
   **DNA-Carbon and ef unitless*

2. <u>Calculate flux (F_z) at depth (z):</u>
   F_z = Flux (euphotic) * $(depth/depth_0)^{(-b)}$ (4)
   Depth = z (500 m and 1000 m where free DNA samples were sequences, see Figure 2 in main text)
   $Depth_0$= net productivity depth, 150 m at Station ALOHA
   b=flux attenuation for organic carbon, 0.89 (2)
   **b is unitless*

3. <u>Source of free DNA to water column at z is flux divergence of value from Step 2 (F_z)</u>
   div = (-b/z)*(F_z)
   **units will be in mg m^{-3} day, which is the same as μg $L^{-1}day^{-1}$*

4. <u>Calculate % of value at 500 m and 1000 m, respectively</u>
   500 m = 0.02 μg L^{-1} (1, 5)
   1000 m = 0.01 μg L^{-1} (1)

<u>Assumptions:</u>

1. Particulate DNA become free DNA by disaggregation rather than degradation on particles (6)
2. Flux conforms to a Martin Curve (4)

<u>References:</u>

1. Linney, M. D., Schvarcz, C. R., Steward, G. F., DeLong, E. F. & Karl, D. M. A method for characterizing dissolved DNA and its application to the North Pacific Subtropical Gyre. Limnol. Oceanogr. Methods.
2. Grabowski, E., Letelier, R. M., Laws, E. A. & Karl, D. M. Coupling carbon and energy fluxes in the North Pacific Subtropical Gyre. Nat. Commun. 10, 1895 (2019).
3. Winn, C. D. & Karl, D. M. Diel nucleic acid synthesis and particulate DNA concentrations: conflicts with division rate estimates by DNA accumulation. Limnol. Oceanogr. 31, 637-645 (1986).
4. Martin, J. H., Knauer, G. A., Karl, D. M. & Broenkow, W. W. VERTEX: carbon cycling in the northeast Pacific. Deep-Sea Res. 34, 267-285 (1987).
5. Brum, J. R. Concentration, production and turnover of viruses and dissolved DNA pools at Stn ALOHA, North Pacific Subtropical Gyre. Aquat. Microb. Ecol. 41, 103-113 (2005).
6. Collins, J. R. et al. The multiple fates of sinking particles in the North Atlantic Ocean. Global Biogeochem. Cycles
